# *N*-Phenyl-*N*-(3-phenyl­prop-2-yn­yl)aniline

**DOI:** 10.1107/S1600536809037866

**Published:** 2009-09-26

**Authors:** Tao Pang, Yi-chong Sun, Jian-ming Zhang

**Affiliations:** a Key Laboratory of Pesticides and Chemical Biology of the Ministry of Education, College of Chemistry, Central China Normal University, Wuhan 430079, People’s Republic of China

## Abstract

In the title compound, C_21_H_17_N, synthesized by a three-component coupling reaction in the presence of copper(I) iodide, the N-bound phenyl rings form a dihedral angle of 72.5 (1)° with each other. Thereare no remarkable inter­actions in the crystal structure.

## Related literature

For the preparation of the title compound, see: Nilsson *et al.* (1992 ▶). For the biological activity of propargylamines and their use as synthetic inter­mediates, see: Bieber & da Silva (2004 ▶); Hattori *et al.* (1993 ▶); Huffman *et al.* (1995 ▶); Konishi *et al.* (1990 ▶).
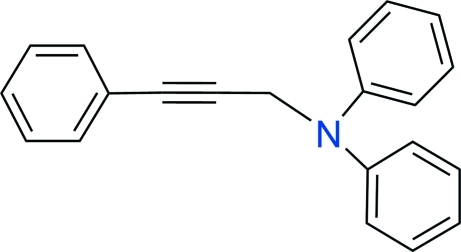


## Experimental

### 

#### Crystal data

C_21_H_17_N
*M* *_r_* = 283.36
Monoclinic, 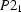

*a* = 11.376 (1) Å
*b* = 5.7287 (5) Å
*c* = 13.409 (1) Å
β = 111.276 (3)°
*V* = 814.30 (12) Å^3^
*Z* = 2
Mo *K*α radiation
μ = 0.07 mm^−1^
*T* = 298 K
0.23 × 0.13 × 0.10 mm

#### Data collection

Bruker SMART CCD diffractometer
Absorption correction: none
5689 measured reflections
1953 independent reflections
1448 reflections with *I* > 2σ(*I*)
*R*_int_ = 0.136

#### Refinement

*R*[*F* ^2^ > 2σ(*F* ^2^)] = 0.055
*wR*(*F* ^2^) = 0.141
*S* = 0.91
1953 reflections
199 parameters
1 restraint
H-atom parameters constrained
Δρ_max_ = 0.15 e Å^−3^
Δρ_min_ = −0.19 e Å^−3^

### 

Data collection: *SMART* (Bruker, 2001 ▶); cell refinement: *SAINT* (Bruker, 2001 ▶); data reduction: *SAINT*; program(s) used to solve structure: *SHELXS97* (Sheldrick, 2008 ▶); program(s) used to refine structure: *SHELXL97* (Sheldrick, 2008 ▶); molecular graphics: *SHELXTL* (Sheldrick, 2008 ▶); software used to prepare material for publication: *SHELXTL*.

## Supplementary Material

Crystal structure: contains datablocks I, global. DOI: 10.1107/S1600536809037866/lx2105sup1.cif

Structure factors: contains datablocks I. DOI: 10.1107/S1600536809037866/lx2105Isup2.hkl

Additional supplementary materials:  crystallographic information; 3D view; checkCIF report

## Figures and Tables

**Fig. 1.**
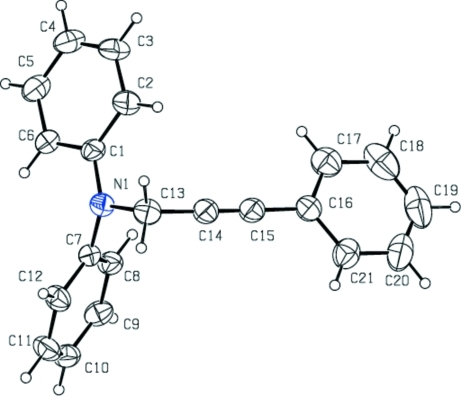
View of the molecule of (I) showing the atom-labelling scheme. Displacement ellipsoids are drawn at the 30% probability level. H atoms are represented by spheres of arbitrary radius.

## supplementary crystallographic information

### Comment

Propargylamines are compounds of interesting biological properties and important synthetic intermediates (Konishi *et al.*, 1990; Huffman *et al.*, 1995; Hattori *et al.*, 1993; Bieber *et al.*, 2004). The reaction which a three component procedure between terminal alkynes, formaldehyde and secondary amines has been extended to some less activated alkynes by the introduction of copper catalysts. Here we report the crystal structure of the title compound (Fig. 1).

In the molecule of the title compound, the N-bound two phenyl rings form a dihedral angle of 72.5 (1)° with each other.

### Experimental

The title compound was synthesized according to the literature procedure of Nilsson *et al.* (1992). Single crystals suitable for X-ray diffraction were prepared by slow evaporation of a solution of the title compound in chloroform : methanol (50 : 1) at room temperature.

### Refinement

All H atoms were initially located in a difference map, but were constrained to an idealized geometry. Constrained bond lengths and isotropic displacement parameters: (C–H = 0.97 Å) and *U*_iso_(H) =1.2 *U*_eq_(C) for methylene, and (C–H = 0.93 Å) and *U*_iso_(H) =1.2*U*_eq_(C) for aromatic H atoms.

### Crystal data

**Table:**

C_21_H_17_N	*F*(000) = 300
*M**_r_* = 283.36	*D*_x_ = 1.156 Mg m^−^^3^
Monoclinic, *P*2_1_	Mo *K*α radiation, λ = 0.71073 Å
Hall symbol: P 2yb	Cell parameters from 1607 reflections
*a* = 11.376 (1) Å	θ = 2.9–22.6°
*b* = 5.7287 (5) Å	µ = 0.07 mm^−^^1^
*c* = 13.409 (1) Å	*T* = 298 K
β = 111.276 (3)°	Block, colorless
*V* = 814.30 (12) Å^3^	0.23 × 0.13 × 0.10 mm
*Z* = 2	

### Data collection

**Table:**

Bruker SMART CCD diffractometer	1448 reflections with *I* > 2σ(*I*)
Radiation source: fine-focus sealed tube	*R*_int_ = 0.136
graphite	θ_max_ = 27.0°, θ_min_ = 1.6°
Detector resolution: 10.0 pixels mm^-1^	*h* = −13→14
φ and ω scans	*k* = −7→7
5689 measured reflections	*l* = −17→10
1953 independent reflections	

### Refinement

**Table:**

Refinement on *F*^2^	Primary atom site location: structure-invariant direct methods
Least-squares matrix: full	Secondary atom site location: difference Fourier map
*R*[*F*^2^ > 2σ(*F*^2^)] = 0.055	Hydrogen site location: difference Fourier map
*wR*(*F*^2^) = 0.141	H-atom parameters constrained
*S* = 0.91	*w* = 1/[σ^2^(*F*_o_^2^) + (0.074*P*)^2^] where *P* = (*F*_o_^2^ + 2*F*_c_^2^)/3
1953 reflections	(Δ/σ)_max_ < 0.001
199 parameters	Δρ_max_ = 0.15 e Å^−^^3^
1 restraint	Δρ_min_ = −0.19 e Å^−^^3^

### Special details

**Table:**

Geometry. All esds (except the esd in the dihedral angle between two l.s. planes) are estimated using the full covariance matrix. The cell esds are taken into account individually in the estimation of esds in distances, angles and torsion angles; correlations between esds in cell parameters are only used when they are defined by crystal symmetry. An approximate (isotropic) treatment of cell esds is used for estimating esds involving l.s. planes.
Refinement. Refinement of F^2^ against ALL reflections. The weighted R-factor wR and goodness of fit S are based on F^2^, conventional R-factors R are based on F, with F set to zero for negative F^2^. The threshold expression of F^2^ > 2sigma(F^2^) is used only for calculating R-factors(gt) etc. and is not relevant to the choice of reflections for refinement. R-factors based on F^2^ are statistically about twice as large as those based on F, and R- factors based on ALL data will be even larger.

### Fractional atomic coordinates and isotropic or equivalent isotropic displacement parameters (Å^2^)

**Table:**

	*x*	*y*	*z*	*U*_iso_*/*U*_eq_	
C1	0.2341 (2)	0.1972 (5)	0.3674 (2)	0.0541 (7)	
C2	0.1912 (3)	0.1537 (7)	0.4505 (2)	0.0716 (9)	
H2	0.1255	0.2420	0.4565	0.086*	
C3	0.2457 (4)	−0.0199 (7)	0.5240 (3)	0.0859 (11)	
H3	0.2158	−0.0466	0.5789	0.103*	
C4	0.3427 (3)	−0.1539 (8)	0.5183 (3)	0.0870 (11)	
H4	0.3790	−0.2695	0.5688	0.104*	
C5	0.3851 (3)	−0.1131 (7)	0.4356 (3)	0.0775 (9)	
H5	0.4500	−0.2039	0.4298	0.093*	
C6	0.3327 (2)	0.0596 (6)	0.3619 (2)	0.0606 (7)	
H6	0.3635	0.0853	0.3074	0.073*	
C7	0.1821 (2)	0.3552 (4)	0.1865 (2)	0.0473 (6)	
C8	0.1297 (2)	0.1628 (5)	0.1253 (2)	0.0555 (7)	
H8	0.0980	0.0420	0.1546	0.067*	
C9	0.1235 (3)	0.1472 (6)	0.0206 (2)	0.0646 (8)	
H9	0.0885	0.0158	−0.0201	0.078*	
C10	0.1692 (3)	0.3265 (6)	−0.0233 (2)	0.0651 (8)	
H10	0.1657	0.3167	−0.0936	0.078*	
C11	0.2198 (3)	0.5184 (6)	0.0370 (3)	0.0690 (8)	
H11	0.2496	0.6407	0.0072	0.083*	
C12	0.2273 (3)	0.5334 (5)	0.1416 (2)	0.0606 (7)	
H12	0.2632	0.6645	0.1821	0.073*	
C13	0.0902 (3)	0.5331 (5)	0.3057 (2)	0.0637 (7)	
H13A	0.0843	0.6719	0.2627	0.076*	
H13B	0.1166	0.5817	0.3799	0.076*	
C14	−0.0356 (3)	0.4259 (6)	0.2741 (2)	0.0611 (7)	
C15	−0.1346 (3)	0.3302 (6)	0.2484 (2)	0.0627 (7)	
C16	−0.2554 (3)	0.2165 (6)	0.2187 (2)	0.0589 (7)	
C17	−0.2702 (4)	0.0104 (7)	0.2656 (3)	0.0808 (9)	
H17	−0.2010	−0.0604	0.3170	0.097*	
C18	−0.3885 (5)	−0.0912 (9)	0.2361 (4)	0.1097 (16)	
H18	−0.3990	−0.2296	0.2681	0.132*	
C19	−0.4903 (5)	0.0132 (13)	0.1594 (5)	0.1160 (19)	
H19	−0.5697	−0.0548	0.1397	0.139*	
C20	−0.4753 (3)	0.2131 (11)	0.1130 (4)	0.1032 (15)	
H20	−0.5446	0.2817	0.0607	0.124*	
C21	−0.3604 (3)	0.3163 (7)	0.1413 (3)	0.0760 (9)	
H21	−0.3518	0.4551	0.1086	0.091*	
N1	0.1865 (2)	0.3795 (4)	0.29364 (18)	0.0548 (6)	

### Atomic displacement parameters (Å^2^)

**Table:**

	*U*^11^	*U*^22^	*U*^33^	*U*^12^	*U*^13^	*U*^23^
C1	0.0509 (14)	0.0643 (16)	0.0386 (12)	−0.0118 (12)	0.0061 (10)	−0.0006 (13)
C2	0.0736 (18)	0.092 (2)	0.0501 (16)	−0.0029 (18)	0.0231 (14)	0.0022 (17)
C3	0.094 (2)	0.114 (3)	0.0463 (16)	−0.006 (2)	0.0216 (17)	0.0205 (19)
C4	0.082 (2)	0.101 (3)	0.061 (2)	0.004 (2)	0.0065 (17)	0.026 (2)
C5	0.0654 (18)	0.086 (2)	0.0677 (19)	0.0086 (16)	0.0085 (15)	0.0125 (19)
C6	0.0503 (14)	0.0747 (19)	0.0496 (15)	−0.0017 (14)	0.0096 (12)	0.0065 (14)
C7	0.0430 (12)	0.0519 (14)	0.0453 (13)	−0.0001 (11)	0.0139 (10)	0.0052 (12)
C8	0.0564 (15)	0.0556 (16)	0.0488 (14)	−0.0085 (12)	0.0120 (11)	0.0017 (13)
C9	0.0681 (18)	0.0638 (18)	0.0508 (15)	−0.0011 (14)	0.0082 (13)	−0.0048 (14)
C10	0.0657 (17)	0.083 (2)	0.0476 (15)	0.0153 (16)	0.0216 (13)	0.0090 (16)
C11	0.0769 (19)	0.0682 (19)	0.071 (2)	−0.0052 (16)	0.0381 (16)	0.0152 (18)
C12	0.0650 (16)	0.0546 (15)	0.0655 (18)	−0.0137 (14)	0.0275 (13)	−0.0015 (15)
C13	0.0715 (18)	0.0593 (16)	0.0621 (17)	0.0007 (15)	0.0265 (14)	−0.0023 (15)
C14	0.0622 (17)	0.0729 (18)	0.0523 (15)	0.0082 (15)	0.0257 (13)	−0.0003 (15)
C15	0.0634 (17)	0.080 (2)	0.0490 (15)	0.0096 (16)	0.0252 (13)	−0.0016 (15)
C16	0.0642 (17)	0.0692 (19)	0.0500 (15)	0.0063 (14)	0.0288 (13)	−0.0089 (15)
C17	0.092 (2)	0.082 (2)	0.072 (2)	−0.0031 (19)	0.0344 (18)	−0.008 (2)
C18	0.141 (4)	0.102 (3)	0.112 (4)	−0.045 (3)	0.077 (4)	−0.033 (3)
C19	0.087 (3)	0.168 (5)	0.110 (4)	−0.052 (3)	0.055 (3)	−0.059 (4)
C20	0.060 (2)	0.149 (5)	0.097 (3)	−0.002 (2)	0.024 (2)	−0.029 (3)
C21	0.0680 (19)	0.085 (2)	0.074 (2)	0.0091 (17)	0.0248 (16)	−0.0135 (18)
N1	0.0555 (12)	0.0624 (13)	0.0476 (12)	−0.0017 (10)	0.0201 (10)	−0.0001 (11)

### Geometric parameters (Å, °)

**Table:**

C1—C2	1.391 (4)	C11—C12	1.377 (4)
C1—C6	1.395 (4)	C11—H11	0.9300
C1—N1	1.405 (4)	C12—H12	0.9300
C2—C3	1.378 (5)	C13—N1	1.459 (3)
C2—H2	0.9300	C13—C14	1.471 (4)
C3—C4	1.368 (5)	C13—H13A	0.9700
C3—H3	0.9300	C13—H13B	0.9700
C4—C5	1.379 (5)	C14—C15	1.185 (4)
C4—H4	0.9300	C15—C16	1.441 (5)
C5—C6	1.372 (5)	C16—C17	1.377 (5)
C5—H5	0.9300	C16—C21	1.390 (4)
C6—H6	0.9300	C17—C18	1.386 (6)
C7—C8	1.374 (4)	C17—H17	0.9300
C7—C12	1.377 (4)	C18—C19	1.376 (8)
C7—N1	1.425 (3)	C18—H18	0.9300
C8—C9	1.383 (4)	C19—C20	1.344 (7)
C8—H8	0.9300	C19—H19	0.9300
C9—C10	1.377 (5)	C20—C21	1.358 (5)
C9—H9	0.9300	C20—H20	0.9300
C10—C11	1.362 (5)	C21—H21	0.9300
C10—H10	0.9300		
			
C2—C1—C6	117.6 (3)	C7—C12—C11	120.3 (3)
C2—C1—N1	122.7 (3)	C7—C12—H12	119.8
C6—C1—N1	119.6 (2)	C11—C12—H12	119.8
C3—C2—C1	120.2 (3)	N1—C13—C14	114.0 (2)
C3—C2—H2	119.9	N1—C13—H13A	108.7
C1—C2—H2	119.9	C14—C13—H13A	108.7
C4—C3—C2	121.8 (3)	N1—C13—H13B	108.7
C4—C3—H3	119.1	C14—C13—H13B	108.7
C2—C3—H3	119.1	H13A—C13—H13B	107.6
C3—C4—C5	118.3 (3)	C15—C14—C13	177.1 (3)
C3—C4—H4	120.9	C14—C15—C16	178.9 (3)
C5—C4—H4	120.9	C17—C16—C21	118.7 (3)
C6—C5—C4	120.9 (3)	C17—C16—C15	121.6 (3)
C6—C5—H5	119.5	C21—C16—C15	119.7 (3)
C4—C5—H5	119.5	C16—C17—C18	119.8 (4)
C5—C6—C1	121.1 (3)	C16—C17—H17	120.1
C5—C6—H6	119.5	C18—C17—H17	120.1
C1—C6—H6	119.5	C19—C18—C17	119.9 (5)
C8—C7—C12	118.9 (2)	C19—C18—H18	120.1
C8—C7—N1	122.0 (2)	C17—C18—H18	120.1
C12—C7—N1	119.1 (2)	C20—C19—C18	120.2 (4)
C7—C8—C9	120.6 (3)	C20—C19—H19	119.9
C7—C8—H8	119.7	C18—C19—H19	119.9
C9—C8—H8	119.7	C19—C20—C21	120.8 (4)
C10—C9—C8	119.9 (3)	C19—C20—H20	119.6
C10—C9—H9	120.1	C21—C20—H20	119.6
C8—C9—H9	120.1	C20—C21—C16	120.6 (4)
C11—C10—C9	119.5 (3)	C20—C21—H21	119.7
C11—C10—H10	120.3	C16—C21—H21	119.7
C9—C10—H10	120.3	C1—N1—C7	120.1 (2)
C10—C11—C12	120.8 (3)	C1—N1—C13	118.9 (2)
C10—C11—H11	119.6	C7—N1—C13	114.6 (2)
C12—C11—H11	119.6		
			
C6—C1—C2—C3	0.1 (4)	C14—C15—C16—C21	90 (18)
N1—C1—C2—C3	−176.6 (3)	C21—C16—C17—C18	−0.8 (4)
C1—C2—C3—C4	0.1 (5)	C15—C16—C17—C18	178.9 (3)
C2—C3—C4—C5	−0.6 (6)	C16—C17—C18—C19	0.6 (5)
C3—C4—C5—C6	0.9 (6)	C17—C18—C19—C20	0.1 (6)
C4—C5—C6—C1	−0.8 (5)	C18—C19—C20—C21	−0.6 (6)
C2—C1—C6—C5	0.2 (4)	C19—C20—C21—C16	0.4 (6)
N1—C1—C6—C5	177.1 (3)	C17—C16—C21—C20	0.3 (5)
C12—C7—C8—C9	0.6 (4)	C15—C16—C21—C20	−179.3 (3)
N1—C7—C8—C9	178.2 (2)	C2—C1—N1—C7	−148.9 (3)
C7—C8—C9—C10	−0.5 (4)	C6—C1—N1—C7	34.4 (3)
C8—C9—C10—C11	−0.2 (4)	C2—C1—N1—C13	1.4 (4)
C9—C10—C11—C12	1.0 (4)	C6—C1—N1—C13	−175.3 (3)
C8—C7—C12—C11	0.1 (4)	C8—C7—N1—C1	50.4 (3)
N1—C7—C12—C11	−177.5 (3)	C12—C7—N1—C1	−132.0 (3)
C10—C11—C12—C7	−0.9 (4)	C8—C7—N1—C13	−101.1 (3)
N1—C13—C14—C15	9(6)	C12—C7—N1—C13	76.4 (3)
C13—C14—C15—C16	136 (16)	C14—C13—N1—C1	−74.1 (3)
C14—C15—C16—C17	−89 (18)	C14—C13—N1—C7	77.8 (3)

## References

[bb1] Bieber, L. W. & da Silva, M. F. (2004). *Tetrahedron Lett.* **45**, 8281–8283.

[bb2] Bruker (12001). *SMART* and *SAINT* Bruker AXS Inc., Madison, Wisconsin, USA.

[bb4] Hattori, K., Miyata, M. & Yamamoto, H. (1993). *J. Am. Chem. Soc.* **115**, 1151–1152.

[bb5] Huffman, M. A., Yasuda, N., DeCamp, A. E. & Grabowski, E. J. J. (1995). *J. Org. Chem.* **60**, 1590–1594.

[bb6] Konishi, M., Ohkuma, H., Tsuno, T., Oki, T., VanDuyne, G. D. & Clardy, J. (1990). *J. Am. Chem. Soc.* **112**, 3715–3716.

[bb7] Nilsson, B. M., Vargas, H. M., Ringdahl, B. & Hacksell, U. (1992). *J. Med. Chem.* **35**, 285–294. 10.1021/jm00080a013 1732545

[bb8] Sheldrick, G. M. (2008). *Acta Cryst.* A**64**, 112–122. 10.1107/S0108767307043930 18156677

